# TiO_2_‐Coated Interlayer‐Expanded MoSe_2_/Phosphorus‐Doped Carbon Nanospheres for Ultrafast and Ultralong Cycling Sodium Storage

**DOI:** 10.1002/advs.201801222

**Published:** 2018-11-09

**Authors:** Yuyu Wang, Yunxiao Wang, Wenpei Kang, Dongwei Cao, Chenxu Li, Dongxu Cao, Zixi Kang, Daofeng Sun, Rongming Wang, Yuliang Cao

**Affiliations:** ^1^ College of Science School of Materials Science and Engineering China University of Petroleum (East China) Qingdao Shandong 266580 P. R. China; ^2^ College of Chemistry and Molecular Sciences Hubei Key Laboratory of Electrochemical Power Sources Wuhan University Wuhan 430072 P. R. China

**Keywords:** anode materials, interlayer‐expanded MoSe_2_ nanospheres, phosphorus‐doped carbon, sodium‐ion batteries, TiO_2_ coating layer

## Abstract

Based on multielectron conversion reactions, layered transition metal dichalcogenides are considered promising electrode materials for sodium‐ion batteries, but suffer from poor cycling performance and rate capability due to their low intrinsic conductivity and severe volume variations. Here, interlayer‐expanded MoSe_2_/phosphorus‐doped carbon hybrid nanospheres coated by anatase TiO_2_ (denoted as MoSe_2_/P‐C@TiO_2_) are prepared by a facile hydrolysis reaction, in which TiO_2_ coating polypyrrole‐phosphomolybdic acid is utilized as a novel precursor followed by a selenization process. Benefiting from synergistic effects of MoSe_2_, phosphorus‐doped carbon, and TiO_2_, the hybrid nanospheres manifest unprecedented cycling stability and ultrafast pseudocapacitive sodium storage capability. The MoSe_2_/P‐C@TiO_2_ delivers decent reversible capacities of 214 mAh g^−1^ at 5.0 A g^−1^ for 8000 cycles, 154 mAh g^−1^ at 10.0 A g^−1^ for 10000 cycles, and an exceptional rate capability up to 20.0 A g^−1^ with a capacity of ≈175 mAh g^−1^ in a voltage range of 0.5–3.0 V. Coupled with a Na_3_V_2_(PO_4_)_3_@C cathode, a full cell successfully confirms a reversible capacity of 242.2 mAh g^−1^ at 0.5 A g^−1^ for 100 cycles with a coulombic efficiency over 99%.

## 1 Introduction

Sodium‐ion batteries (SIBs) possessing a similar structure characteristics and energy storage mechanism to those of lithium‐ion batteries (LIBs), serving as one of the most promising energy‐storage devices, have attracted our tremendous attention on account of their low cost, abundant, and widespread sodium resources.^1^, ^2^, ^3^, ^4^ Nevertheless, the large ionic radius of an Na^+^ ion (≈1.09 Å), which is 55% larger than that of Li^+^ ion, and its higher molar mass make the sodiation/desodiation kinetics more sluggish, impeding the development and practical application of SIBs.^5^, ^6^, ^7^ For these reasons, numerous effort including development of appropriate anode materials has been taken to satisfy the demands of decent specific capacity, long cycle life, and excellent rate performance for stationary energy storage.^8^, ^9^, ^10^

MoSe_2_, a representative type of layered transition metal dichalcogenide (TMD) material, exhibits high theoretical capacity, large interlayer spacing, and small bandgap, and thus has been proved to be a good candidate anode material for SIBs.^11^, ^12^, ^13^, ^14^, ^15^ Unfortunately, it still suffers from rapid cycling capacity degradation caused by large volume change and slow kinetics during the sodiation/desodiation process. According to previous research,^16^, ^17^, ^18^, ^19^ construction of rational nanostructures and formation of carbon‐based composites are effective approaches. Various nanostructures, including MoSe_2_@porous hollow carbon spheres,^20^ carbon‐stabilized interlayer‐expanded MoSe_2_ nanosheets,^21^ and fullerene‐like MoSe_2_ nanoparticles‐embedded CNT balls,^22^ have been explored to enhance the electrochemical performance of MoSe_2_. On the other hand, the interlayer‐expanded structures are very critical, which can provide efficient ion migration channels, facilitating to enhance charge/discharge kinetics. Sun's group successfully synthesized interlayer‐expanded MoSe_2_@hollow carbon nanosphere (HCNS) materials, which exhibited a specific capacity of 471 mAh g^−1^ after 1000 cycles at 3.0 A g^−1^.^23^ These results confirm that sodium storage performance can be enhanced through rational nanostructure engineering. However, in view of the long cycle life for practical applications, the serious volume changes during charge and discharge processes remain challenging and need to be further alleviated.

As one of the typical insertion‐type electrode materials for secondary batteries, TiO_2_ possesses reasonable insertion potential and negligible change in volume, showing good structural stability and long cycle life.^24^, ^25^, ^26^, ^27^, ^28^, ^29^ Although its theoretical specific capacity is fairly low, it can serve as an effective protective layer for other electrode materials with severe volume expansion, such as metal oxides or metal sulfides through smart hybridization.^30^, ^31^, ^32^, ^33^, ^34^, ^35^ Such an approach can effectively ensure the specific capacity and achieve a long cycle life attributed to that the rigid TiO_2_ layer could tolerate the interior volume variation of metal oxides or metal sulfides. For instance, Wang and co‐workers reported porous hollow α‐Fe_2_O_3_@TiO_2_ core–shell nanospheres.^30^ When used as anode materials in SIBs, these hybrid hierarchical nanospheres delivered a high reversible capacity of 267 mAh g^−1^ at 100 mA g^−1^ with an excellent cycling stability up to 300 cycles.

In this work, we report an elaborate anode, consisted of interlayer‐expanded MoSe_2_/phosphorus‐doped carbon nanospheres composite and anatase TiO_2_ coating (denoted as MoSe_2_/P‐C@TiO_2_). In a facile hydrolysis reaction in acidic medium, TiO_2_ layer was coated on PPy‐PMo_12_ precursor nanospheres, followed by a confined selenization process, MoSe_2_/P‐C@TiO_2_ nanospheres can be obtained by inheriting the morphology of the PPy‐PMo_12_ precursor. Compared to previous reports on MoSe_2_‐based composite electrodes, our MoSe_2_‐based hybrid has the following advantages: (i) the phosphorus‐doped carbon transformed from the PPy‐PMo_12_ precursor, can increase the conductivity of nanocomposite and provide more active sites between Na^+^ and the electrolyte to improve the charge transfer efficiency; (ii) the expanded interlayer spacing of MoSe_2_ can decrease the ion diffusion resistance and facilitate the fast Na^+^ insertion/extraction reaction kinetics; (iii) the coated TiO_2_ layer plays an essential role in alleviating the volume changes upon sodiation/desodiation process, thus preserving the electrode integrity; and (iv) the synergistic effect of MoSe_2_, phosphorus‐doped carbon, and TiO_2_ can enhance the electrochemical performance for SIBs. Benefiting from the unique configuration of the as‐fabricated MoSe_2_/P‐C@TiO_2_ nanospheres, when used as an anode material for SIBs, it exhibits ultralong cycle stability, superior specific capacity, and exceptional rate capability. Additionally, this hybrid structure manifests a relatively small irreversible capacity loss with a high initial columbic efficiency (ICE) of 81.3%, which further enable it a feasible anode for practical full cells.

## 2 Results and Discussion

**Figure 1:**
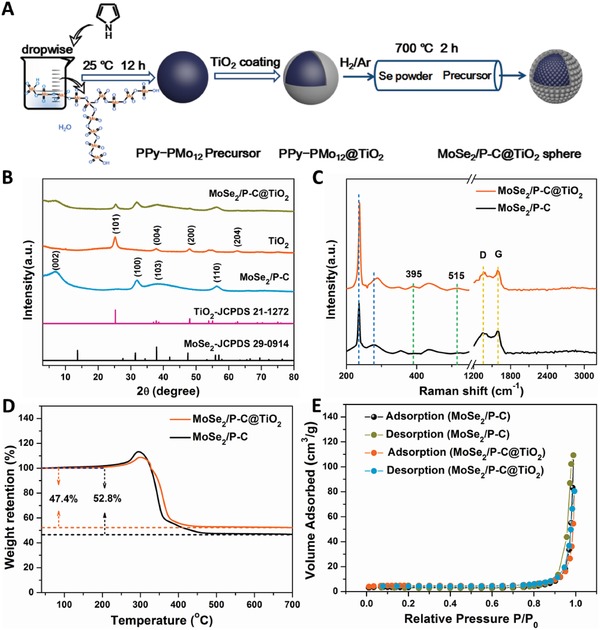
A) Schematic illustration of the synthesis process for the spherical MoSe_2_/P‐C@TiO_2_ nanocomposite. B) XRD patterns. C) Raman spectra. D) TGA curves. E) N_2_ adsorption/desorption isotherm of MoSe_2_/P‐C@TiO_2_ hybrid nanospheres and MoSe_2_/P‐C nanospheres.

The synthesis process of MoSe_2_/P‐C@TiO_2_ nanospheres is schematically illustrated in **Figure** **1**A. Briefly, the uniform PPy‐PMo_12_ nanospheres are first synthesized through a polymerization reaction of phosphomolybdic acid and pyrrole forming polypyrrole‐phosphomolybdic acid (PPy‐PMo_12_). Then a layer of TiO_2_ nanoparticles is grown on the PPy‐PMo_12_ nanospheres through a deposition process in chemical bath. Finally, MoSe_2_/P‐C@TiO_2_ nanospheres are successfully obtained after calcination with Se powder in a tube furnace. To estimate the influence of P‐C and TiO_2_ coating, MoSe_2_/P‐C and MoSe_2_@TiO_2_ composites were also prepared following a similar process.

Figure 1B shows the X‐ray diffraction (XRD) patterns of as‐obtained MoSe_2_/P‐C, TiO_2_, and MoSe_2_/P‐C@TiO_2_ nanospheres. The diffraction peaks at 25.28°, 37.80°, 48.05°, and 62.68° can be assigned to (101), (004), (200), and (204) planes of anatase TiO_2_ (JCPDS No. 21‐1272). Three characteristic peaks at 31.42°, 37.88°, and 55.92° are consistent with the planes of (100), (103), and (110) for the hexagonal MoSe_2_ phase (JCPDS No. 29‐0914). In addition, a peak at ≈7.04° can be observed, which corresponds to expanded (002) plane, exhibiting an obvious shift of 6.66° compared with the JCPDS data. According to the Bragg equation, the interlayer spacing is calculated to be 1.26 nm, indicating formation of an expanded interlayer, which may be attributed to the intercalation of in situ carbon between the interlayers^23^, ^36^ during the PPy‐PMo_12_ precursor calcination with Se powder at 700 °C. Raman spectra were used to further characterize the MoSe_2_, TiO_2_, and carbon materials in the composite. In Raman spectrum of the MoSe_2_/P‐C@TiO_2_ (Figure 1C), there are two weak peaks at ≈395 and 515 cm^−1^, which symbolize the B^1^_g_ and A^1^_g_ modes of the anatase phase of the TiO_2_.^37^ Meanwhile, a strong peak at 237.6 cm^−1^ is the A^1^_g_ mode originating from the out‐of‐plane vibration of the Mo—Se band and another peak at ≈289.8 cm^−1^ belongs to the E^1^_2g_ mode originating from the Mo—Se in‐plane vibration.^14^, ^19^ Compared to the MoSe_2_/P‐C, slight shifts of the corresponding peaks occurred due to the surface strain that may be caused by the TiO_2_ coating.^38^ In addition, two peaks around 1360 and 1591 cm^−1^ are observed, corresponding to the D band for defected and disordered carbon and the G band derived from the vibration of sp^2^ carbon atoms, respectively.^14^, ^19^, ^39^ The *I*_D_/*I*_G_ intensity ratio is 0.92, higher than 0.90 in the MoSe_2_/P‐C, indicating a more defected and disordered structure in the carbon component of MoSe_2_/P‐C@TiO_2_.^39^ As roughly verified by thermogravimetric analysis (TGA), as shown in Figure 1D, the mass contents of carbon, MoSe_2_, and TiO_2_ in the composite are 15.11, 74.36, and 10.53%, respectively. And the detailed calculating process is added in the Supporting Information. Moreover, the MoSe_2_/P‐C@TiO_2_ nanospheres exhibit a specific Brunauer–Emmett–Teller (BET) surface area of 14.18 m^2^ g^−1^ (Figure 1E), suggesting there are sufficient active sites for the electrolyte contact when used as anode material in SIBs.

**Figure 2:**
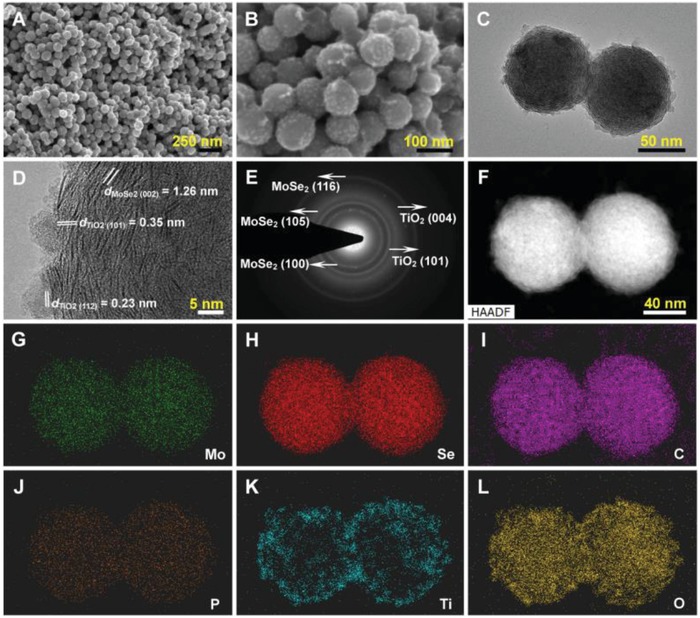
Morphology and structure of the MoSe_2_/P‐C@TiO_2_ hybrid nanospheres. A,B) FESEM images. C) TEM images. D) HRTEM image. E) The corresponding SAED pattern. F–L) HAADF‐STEM image and the corresponding EDX mappings of Mo, Se, C, P, Ti, and O. The scale bar in (F) also applies to (G)–(L).

Field emission scanning electron microscope (FESEM) and transmission electron microscope (TEM) images offer insights into the morphology and microstructure of the product. A panoramic view of the product (**Figure** **2**A) indicates that it is composed of uniform nanospheres with size ≈100 nm, which is consistent with the PPy‐PMo_12_ precursor and MoSe_2_/P‐C (Figure S1, Supporting Information). This result reveals that the MoSe_2_/P‐C@TiO_2_ can still maintain the nanosphere without any aggregation or collapse after TiO_2_ coating and even high‐temperature calcination. Compared with the smooth surface of the PPy‐PMo_12_ precursor and MoSe_2_/P‐C, the MoSe_2_/P‐C@TiO_2_ possesses a rough surface with assembled nanoparticles (Figure 2B), suggesting the successful coating of the TiO_2_ layer. It should be noted that the agglomeration of MoSe_2_@TiO_2_ occurs (Figure S2, Supporting Information), indicating the importance of in situ P—C on the sphere structure preservation. As can be seen from Figure 2C, the layered structure and well‐defined TiO_2_ layers on the surface of the nanospheres can be clearly observed in the low‐magnification TEM image. A typical high‐resolution TEM (HRTEM) image is shown in Figure 2D, from which the expanded (002) lattice spacing (≈1.26 nm) of MoSe_2_ can be clearly observed and is consistent with the XRD results. The increased interlayer spacing means lower ion diffusion resistance and more available and accessible active surface area, promoting the rate capability when used as an SIB anode.^19^, ^40^ Besides, two distinct lattice fringes at the edge of nanospheres with spacings of 0.35 and 0.23 nm can be observed, respectively, in well agreement with the (101) and (112) planes of anatase TiO_2_. The selected‐area electron diffraction (SAED) pattern (Figure 2E) shows multiple diffraction rings, confirming the formation of a polycrystalline structure. As shown in Figure 2F–L and Figure S3 (Supporting Information), the high‐angle annular dark‐field scanning TEM (HAADF‐STEM) image and the corresponding energy‐dispersive X‐ray spectrum (EDX) elemental mappings unambiguously identify the Mo, Se, C, and P elements covering the entire spherical structure, while Ti and O elements are mainly dispersed on the surface of the sphere, revealing that the architecture has ingeniously integrated features of the hybrid nanostructures.

**Figure 3:**
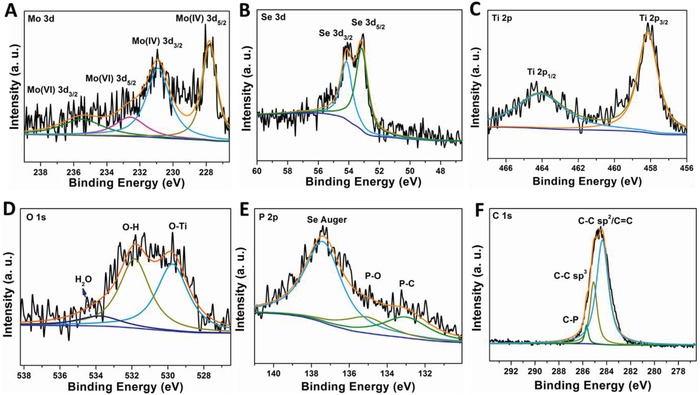
XPS spectra of the MoSe_2_/P‐C@TiO_2_ composite. A–F) High‐resolution XPS spectra for Mo 3d, Se 3d, Ti 2p, O 1s, P 2p, and C 1s.

X‐ray photoelectron spectroscopy (XPS) spectra (**Figure** **3**) were recorded to further examine the surface information and oxidation states of the chemical elements in the MoSe_2_/P‐C@TiO_2_ nanospheres. Six elements, Mo, Se, Ti, O, C, and P, can be clearly seen from the survey spectrum in Figure S4 (Supporting Information). The Mo 3d core level spectrum is shown in Figure 3A, which can be fitted into four peaks at 227.8, 230.9, 232.6, and 235.5 eV. The characteristic peaks at 227.8 and 230.9 eV can be attributed to Mo^4+^ 3d_3/2_ and Mo^4+^ 3d_5/2_, respectively.^23^ In addition, the peaks at 232.6 and 235.5 eV represent the appearance of Mo^6+^ due to the partial oxidization of MoSe_2_.^41^ In the Se 3d spectrum (Figure 3B), there are two peaks located at 53.1 and 54.0 eV, which are assigned, respectively, to the Se 3d_5/2_ and Se 3d_3/2_.^42^ Figure 3C depicts the Ti 2p core level spectrum with two peaks centered at 458.1 eV for Ti 2p_3/2_ and 463.9 eV for Ti 2p_5/2_.^43^ The distance between the Ti 2p_1/2_ and Ti 2p_3/2_ core levels is 5.8 eV, suggesting a normal state of Ti^4+^ in the anatase TiO_2_.^33^, ^44^ As shown in the O 1s spectrum (Figure 3D), the binding energy of Ti—O, O—H, and H_2_O is observed at 529.8, 531.9, and 533.8 eV, respectively.^45^, ^46^ These results indicate the successful coating of the anatase TiO_2_ phase. The XPS spectrum of the P 2p core‐level (Figure 3E) displays two peaks at 133.0 and 135.1 eV, which are related to P—C and P=O bonds, respectively.^11^, ^47^ The C 1s spectrum (Figure 3F) could be divided into three peaks, which are located at 284.3, 285.1, and 285.7 eV, corresponding to sp^2^ C, sp^3^ C, and C—P bonds, respectively.

**Figure 4:**
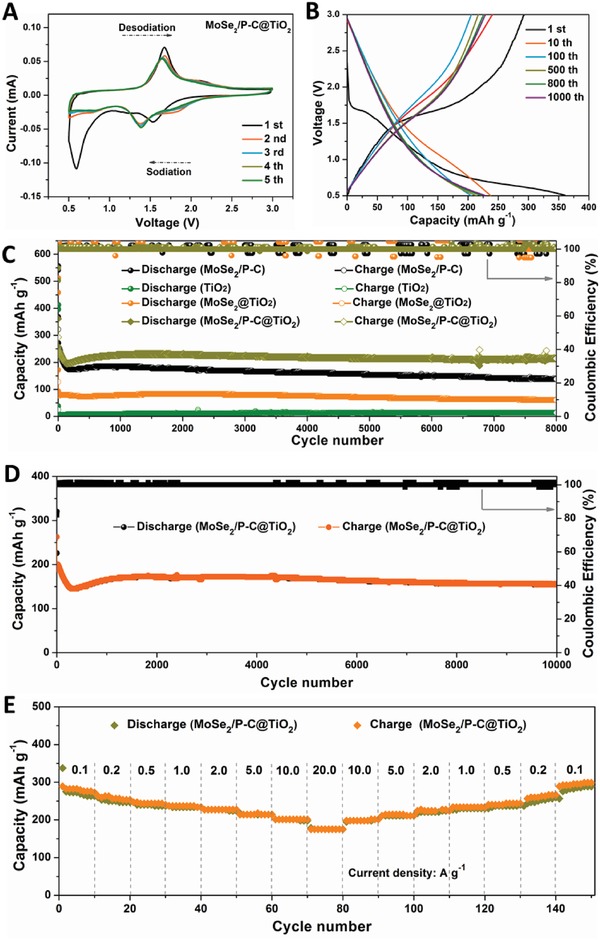
Electrochemical performance of the MoSe_2_/P‐C@TiO_2_ electrode. A) CV curves at a scan rate of 0.1 mV s^−1^. B) Typical charge–discharge profiles at 5.0 A g^−1^. C,D) Cycling performance at 5.0 and 10.0 A g^−1^. E) Rate cycling behavior.

The successful synthesis of the MoSe_2_/P‐C@TiO_2_ hybrid nanospheres for a superior SIB anode material is confirmed from the excellent electrochemical behavior (**Figure** **4**). Figure 4A shows the cyclic voltammetry (CV) curves of MoSe_2_/P‐C@TiO_2_ anode in a voltage range of 0.5–3.0 V versus Na/Na^+^ at a scan rate of 0.1 mV s^−1^ for the initial five cycles. During the first discharge process, three main cathodic peaks at 1.53, 0.60, and 0.50 V can be observed. Compared with CV curves of the MoSe_2_/P‐C and TiO_2_ (Figure S5, Supporting Information), the peak at 1.53 V corresponds to the Na^+^ intercalation into anatase TiO_2_ phase.^48^ The reduction peak at 0.60 eV can be attributed to the Na^+^ insertion into the interlayer of MoSe_2_ and formation of Na*_x_*MoSe_2_, and the other peak at 0.50 eV is assigned to conversion reaction from Na*_x_*MoSe_2_ to Mo metal and Na_2_Se with the formation of a solid electrolyte interphase (SEI) layer.^23^, ^49^ Accordingly, the oxidation peaks at 1.67 and 2.10 V are associated with the oxidation of Mo to MoSe_2_ and Na*_x_*TiO_2_ to TiO_2_, respectively. After the first cycle, the following CV curves are almost overlapped and the reduction peaks move to higher potential, indicating the good reversibility of MoSe_2_/P‐C@TiO_2_ anode during the repeated sodiation/desodiation processes. The main peak locating at 1.38 V should be attributed to the reactions of Na^+^ insertion, the interlayer of MoSe_2_, and subsequent reduction into Mo and Na_2_Se.

Figure 4B exhibits the galvanostatic discharge–charge curves of MoSe_2_/P‐C@TiO_2_ composite electrode at 5.0 A g^−1^ in the voltage range of 0.5–3.0 V, which are well consistent with the CV results. The initial charge and discharge capacities are ≈293.3 and 360.8 mAh g^−1^, respectively, giving an ICE of 81.3%. As the cycles proceed, the charge and discharge capacities from 10th to 100th cycles show a trend of decrease followed by a slight increase in subsequent cycles. This result is further demonstrated by a galvanostatic charge–discharge cycling test, as shown in Figure 4C,D. The reversible discharge capacities for the 500th, 800th, and 1000th cycles are 214, 223, and 229 mAh g^−1^ with average CEs up to ≈100%, showing an outstanding cycling stability. The cycling performance of the MoSe_2_/P‐C and TiO_2_ is also carried out at the current of 5.0 A g^−1^ (Figure 4C), which clearly demonstrates the effects of the TiO_2_ modified MoSe_2_/P‐C in improving the sodium storage performance. It is found that although the reversible capacity of TiO_2_ is noticeably low in the voltage range of 0.5–3.0 V, its cycling stability is clearly better than the other two materials. The MoSe_2_/P‐C electrode shows higher discharge capacities than those of MoSe_2_/P‐C@TiO_2_ in the initial 40 cycles. After 40 cycles, it exhibits a rapid decrease and stabilizes the discharge capacity of only 138 mAh g^−1^ after 8000 cycles with a low capacity retention of 50.7% in comparison with the value for the second cycle. And the capacities of MoSe_2_@TiO_2_ are lower and a capacity of 60.5 mAh g^−1^ can be maintained after 8000 cycles. In contrast, the reversible capacity of MoSe_2_/P‐C@TiO_2_ electrode decreases slightly in the initial 243 cycles because of the polarization when cycled at high current and delivers a little increase capacity attributed to progressive kinetic activation in the electrode.^12^, ^31^, ^40^, ^48^ After those, a stable capacity of 214 mAh g^−1^ can be delivered after 8000 cycles, giving a capacity retention of 82.9%. This result indicates that MoSe_2_/P‐C@TiO_2_ electrode, as expected, exhibits a synthetic effect combining the advantages for the individual components. Moreover, long cycling life can be verified as shown in Figure 4D. Even at a high current density of 10.0 A g^−1^, the discharge capacity of 154 mAh g^−1^ can be maintained over 10 000 cycles with CEs as high as 100%, delivering a capacity retention of 68.1%.

As expected, the MoSe_2_/P‐C@TiO_2_ hybrid electrode manifests an excellent high rate capability, as indicated in Figure 4E. At current densities of 0.1, 0.2, 0.5, 1.0, 2.0, and 5.0 A g^−1^, the reversible capacities of the hybrid nanospheres are ≈270, 250, 240, 236, 228, and 214 mAh g^−1^, respectively. Even at the high current densities of 10.0 and 20.0 A g^−1^, the hybrid can still deliver capacities as high as 202 and 175 mAh g^−1^, respectively. This high‐rate sodium‐storage capability exceeds those of many other MoS_2_/MoSe_2_‐based nanostructures such as MoS_2_:C nanotube, MoS_2_/C hybrid, MoS_2_ nanoflowers, PEO‐MoS_2_ nanocomposites, C‐MoSe_2_/rGO composite, and few‐layered and carbon‐modified MoSe_2_ nanotube (Figure S6, Supporting Information). More importantly, when the rate is gradually decreased back to 0.1 A g^−1^, the capacity can recover to 293 mAh g^−1^, which could originate from the reactivation process caused by high‐rate sodiation.

**Figure 5:**
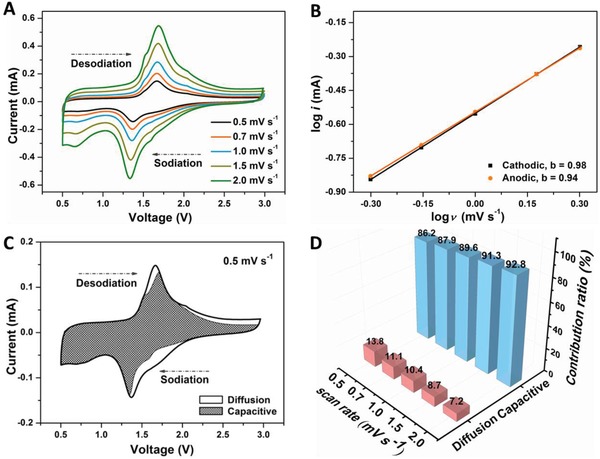
Kinetics analysis of the sodium storage behavior for the MoSe_2_/P‐C@TiO_2_ electrode. A) CV curves at different scan rates. B) *b*‐value analysis based on the relationship between the peak currents and the scan rates. C) Capacitive (shade region) and diffusion‐controlled charge storage contributions for the CV curve at scan rate of 0.5 mV s^−1^. D) Contribution ratio of the capacitive and diffusion‐controlled charges at different scan rates.

To explore the underlying reason of the long cycling and ultrafast sodium storage of the MoSe_2_/P‐C@TiO_2_ electrode, the redox pseudocapacitive contribution in the hybrid electrode was examined by separating the capacitive capacity and the diffusion‐controlled capacity. The reaction kinetics of the MoSe_2_/P‐C@TiO_2_ nanospheres with Na^+^ ions can be investigated by CV profiles at different sweep rates (ν) from 0.5 to 2.0 mV s^−1^. As displayed in **Figure** **5**A, all the CV curves have similar peak shapes except for the corresponding shift during Na^+^ insertion/extraction processes. The total stored charges contributed by diffusion‐controlled or/and capacitive contributions can be divided using the power law *i* = *aν^b^*,^24^, ^40^, ^49^, ^50^ where ν is the scan rate, and both *a* and *b* are adjustable parameters. Generally, *b* = 0.5 indicates a diffusion‐controlled process, and *b* = 1.0 represents capacitive‐contributed charge storage. Figure 5B shows the slope of the corresponding log (ν) versus log (*i*) plots, in which the *b*‐values are 0.98 and 0.94 for cathodic and anodic peaks, respectively. These values suggest that the two mechanisms coexist in the sodiation/desodiation process, which is mainly controlled by the capacitive process. Moreover, the contributions from the two mechanisms can be estimated from the formula *i*(*V*) = *k*_1_ν + *k*_2_ν^1/2^ at a fixed potential.^11^, ^49^, ^50^ As shown in Figure 5C, 86.2% of the total capacity is attributed to the capacitive contribution at a scan rate of 0.5 mV s^−1^. Along with the sweep rate increases, the capacitance contribution gradually increases, reaching a value of 92.8% at 2.0 mV s^−1^ (Figure 5D). The kinetics analyses obviously manifest the gradual increase of capacity contribution, where the fast electrochemical kinetics of involved surficial reactions contribute to the high rate capability. The improved Na^+^‐reaction kinetics of the MoSe_2_/P‐C@TiO_2_ anode material are further supported by the lower *R*_ct_ than that of the MoSe_2_/P‐C electrode (Figure S7A, Supporting Information) and the decreasing charge‐transfer resistance upon cycling (Figure S7B, Supporting Information). Postmortem study verifies that the structure of the MoSe_2_/P‐C@TiO_2_ hybrid spheres can be well retained after 1000 cycles at a current density of 5.0 A g^−1^ (Figure S8, Supporting Information). This confirms TiO_2_ coating can relieve structural collapse and promote ultralong cycling stability.

In view of the superior sodium‐storage performance of MoSe_2_/P‐C@TiO_2_ in a half cell, full cells are further assembled with homemade Na_3_V_2_(PO4)_3_@C (NVP@C) as the cathode material to evaluate its potential for practical application. The homemade NVP@C was synthesized by a hydrothermal assisted sol‐gel method,^51^ which has good phase purity and crystal structure as shown in Figure S9A (Supporting Information). When used as cathode material for a half cell, it exhibits a capacity of 80.8 mAh g^−1^ at 200 mA g^−1^ after 100 cycles and shows a flat voltage plateau around 3.4 V (Figure S9B,C, Supporting Information), which is consistent with previous reports.^9^, ^26^, ^52^ For a sodium‐ion full battery, a discharge voltage plateau around 1.85 V can be seen from the galvanostatic discharge–charge curves at 0.5 A g^−1^ in the voltage range of 0.5–3.0 V (Figure S10A, Supporting Information). Figure S10B (Supporting Information) shows the cycling stability of the MoSe_2_/P‐C@TiO_2_//NVP@C full cells at current density of 500 mA g^−1^. A reversible capacity of 242.2 mAh g^−1^ can be maintained after 100 cycles with the CE over 99%, suggesting a robust cyclability of MoSe_2_/P‐C@TiO_2_ when applied into full cells.

## 3 Conclusion

In conclusion, a facile self‐templating strategy has been developed to synthesize PPy‐PMo_12_@TiO_2_ nanospheres using PPy‐PMo_12_ as the precursor via a hydrolysis reaction. Through a subsequent simultaneous selenization–carbonization process, MoSe_2_/P‐C@TiO_2_ hybrid nanospheres can be easily achieved. Benefiting from the unique structural and multicompositional features, the MoSe_2_/P‐C@TiO_2_ hybrid nanospheres provide superior sodium‐storage performance based on high capacity, excellent cycling stability, and exceptional rate capability. Specifically, the MoSe_2_/P‐C@TiO_2_ hybrid nanospheres deliver a reversible capacity of 214 mAh g^−1^ at 5.0 A g^−1^ for 8000 cycles and 154 mAh g^−1^ at 10.0 A g^−1^ for 10000 cycles as well as an ultrahigh rate capability up to 20.0 A g^−1^ with a capacity of 175 mAh g^−1^. This work could open up a new avenue to design and prepare complex multicompositional hybrids toward various applications.

## 4 Experimental Section

*Synthesis of PPy‐PMo_12_ Precursor*: According to a previous report,^47^ the synthetic method is as follows. Pyrrole solution (420 µL in 25 mL of absolute ethanol) was added dropwise to an aqueous solution of phosphomolybdic acid (H_3_PMo_12_O_40_·*x*H_2_O, 2.19 g in 100 mL of deionized H_2_O). After the mixture was continuously stirred at room temperature for 12 h and then aged for 24 h, the product was obtained through centrifugation, washed with deionized H_2_O and absolute ethanol several times, and then dried at 60 °C. The PPy‐PMo_12_ precursor was collected for further use.

*Synthesis of MoSe_2_/P‐C@TiO_2_ Nanospheres*: In a typical procedure,^35^ first, 200 mg of the above PPy‐PMo_12_ precursor was dispersed in 96.7 mL 0.1 m HCl solution which contains 2 g dihydroxybis (ammonium lactato) titanium (IV) (50 wt% solution in H_2_O) under magnetic stirring at room temperature for 2 h. The PPy‐PMo_12_@TiO_2_ nanoparticles were collected by centrifugation, washed with deionized H_2_O and absolute ethanol several times, and then dried at 60 °C. The PPy‐PMo_12_@TiO_2_ (100 mg) was placed in a porcelain boat at the downstream side of the furnace with 200 mg of Se power placed another boat at the upstream side in the tube furnace. Eventually, the MoSe_2_/P‐C@TiO_2_ nanospheres were obtained after heated at 700 °C with a scan rate of 1 °C min^−1^ in a H_2_/Ar flow for 2 h. The MoSe_2_/P‐C nanospheres were prepared in the same condition except the TiO_2_ coating. And TiO_2_ was prepared in a similar procedure to MoSe_2_/P‐C@TiO_2_ except the addition of PPy‐PMo_12_ precursor and calcination with Se powder. MoSe_2_@TiO_2_ was prepared using a similar procedure to MoSe_2_/P‐C@TiO_2_ except the calcination of PPy‐PMo_12_ at 600 °C in air before calcination with Se powder in order to remove the carbon source of PPy.

*Materials Characterizations*: XRD patterns were performed on a Rigaku UItima IV X‐Ray Diffractometer. FESEM images were recorded by a scanning electron microscope (Philips XL30 FEG SEM). TEM, HRTEM, and HAADF‐STEM images were collected on FEI Talos F200X TEM at 200 kV. Raman spectra were carried out on a HORIBA Evolution Ramanscope with the excitation wavelength of 532 nm. TGA was carried out using PerkinElmer TGA 7 in an air atmosphere with a heating rate of 10 °C min^−1^. XPS was measured by Thermo ESCALAB 250 surface analysis system. The N_2_ adsorption–desorption isothermal curve and the BET surface area was calculated via Micro ASAP2020 at 77 K.

*Electrochemical Measurements*: The performances of sodium storage were evaluated by coin cells (CR2032) assembled in an Ar glovebox with a voltage window of 0.5–3.0 V. The sodium metal, glass fiber, and 1.0 m NaCF_3_SO_3_ solution in diethyleneglycoldimethylether (DEGDME) played the parts of counter electrode, separator, and electrolyte, respectively. The working electrode was prepared by forming a H_2_O‐based slurry composed of active material, carbon black, and sodium carboxymethyl cellulose (CMC) with a weight ratio of 6:2:2 on a copper foil. Then the coated foil was dried at 80 °C and cut into 12 mm discs with a loading of ≈1.0 mg cm^−2^. CV curves and electrochemical impedance spectroscopy (EIS) were performed using a Gamry 30115 electrochemical workstation with different scan rates from 0.5 to 2.0 mV s^−1^ and frequencies of 0.1 MHz to 10 mHz. Galvanostatic discharge/charge cycling was carried out using Neware‐5 V10 mA system (Shenzhen Xinwei). For Na‐ion full cells, the cathode was made of homemade Na_3_V_2_(PO_4_)_3_@C, carbon black, and poly(vinylidene fluoride) (PVDF) were mixed in N‐methyl‐2‐pyrrolidone (NMP) with a weight ratio of 8:1:1 to form a slurry which was coated on an aluminum foil.

## Conflict of Interest

The authors declare no conflict of interest.

## Supporting information

Supplementary

## References

[1] L. Xiao , Y. Cao , W. A. Henderson , M. L. Sushko , Y. Shao , J. Xiao , W. Wang , M. H. Engelhard , Z. Nie , J. Liu , Nano Energy 2016, 19, 279.

[2] S. Y. Hong , Y. Kim , Y. Park , A. Choi , N.‐S. Choi , K. T. Lee , Energy Environ. Sci. 2013, 6, 2067.

[3] Y. Liu , N. Zhang , C. Yu , L. Jiao , J. Chen , Nano Lett. 2016, 16, 3321. 27050390 10.1021/acs.nanolett.6b00942

[4] M. D. Slater , D. Kim , E. Lee , C. S. Johnson , Adv. Funct. Mater. 2013, 23, 947.

[5] J. Yang , X. Zhou , D. Wu , X. Zhao , Z. Zhou , Adv. Mater. 2017, 29, 1604108. 10.1002/adma.201604108 27885733

[6] L. Ling , Y. Bai , Z. Wang , Q. Ni , G. Chen , Z. Zhou , C. Wu , ACS Appl. Mater. Interfaces 2018, 10, 5560. 29338166 10.1021/acsami.7b17659

[7] H. Park , J. Kwon , H. Choi , D. Shin , T. Song , X. W. D. Lou , ACS Nano 2018, 12, 2827. 29505231 10.1021/acsnano.8b00118

[8] R. Sun , S. Liu , Q. Wei , J. Sheng , S. Zhu , Q. An , L. Mai , Small 2017, 13, 1701744. 10.1002/smll.201701744 28834239

[9] J. Deng , Q. Gong , H. Ye , K. Feng , J. Zhou , C. Zha , J. Wu , J. Chen , J. Zhong , Y. Li , ACS Nano 2018, 12, 1829. 29397688 10.1021/acsnano.7b08625

[10] W. Kang , Y. Wang , J. Xu , J. Mater. Chem. A 2017, 5, 7667.

[11] F. Niu , J. Yang , N. Wang , D. Zhang , W. Fan , J. Yang , Y. Qian , Adv. Funct. Mater. 2017, 27, 1700522.

[12] M. Zhu , Z. Luo , A. Pan , H. Yang , T. Zhu , S. Liang , G. Cao , Chem. Eng. J. 2018, 334, 2190.

[13] D. Xie , X. Xia , Y. Zhong , Y. Wang , D. Wang , X. Wang , J. Tu , Adv. Energy Mater. 2017, 7, 1601804.

[14] J. Zhang , W. Kang , M. Jiang , Y. You , Y. Cao , T.‐W. Ng , D. Y. W. Yu , C.‐S. Lee , J. Xu , Nanoscale 2017, 9, 1484. 28067399 10.1039/c6nr09166k

[15] Y. N. Ko , S. H. Choi , S. B. Park , Y. C. Kang , Nanoscale 2014, 6, 10511. 25081031 10.1039/c4nr02538e

[16] J. Li , H. Hu , F. Qin , P. Zhang , L. Zou , H. Wang , K. Zhang , Y. Lai , Chem. ‐ Eur. J. 2017, 23, 14004. 28777498 10.1002/chem.201702791

[17] D. Xie , W. Tang , Y. Wang , X. Xia , Y. Zhong , D. Zhou , D. Wang , X. Wang , J. Tu , Nano Res. 2016, 9, 1618.

[18] Z. Zhang , X. Yang , Y. Fu , K. Du , J. Power Sources 2015, 296, 2.

[19] J. Zhang , M. Wu , T. Liu , W. Kang , J. Xu , J. Mater. Chem. A 2017, 5, 24859.

[20] X. Yang , Z. Zhang , Y. Fu , Q. Li , Nanoscale 2015, 7, 10198. 25988607 10.1039/c5nr01909e

[21] Y. Tang , Z. Zhao , Y. Wang , Y. Dong , Y. Liu , X. Wang , J. Qiu , ACS Appl. Mater. Interfaces 2016, 8, 32324. 27933849 10.1021/acsami.6b11230

[22] S. H. Choi , Y. C. Kang , Nanoscale 2016, 8, 4209. 26830784 10.1039/c5nr07733h

[23] H. Liu , H. Guo , B. Liu , M. Liang , Z. Lv , K. R. Adair , X. Sun , Adv. Funct. Mater. 2018, 28, 1707480.

[24] B. Li , B. Xi , Z. Feng , Y. Lin , J. Liu , J. Feng , Y. Qian , S. Xiong , Adv. Mater. 2018, 30, 1705788. 10.1002/adma.201705788 29334133

[25] J. Ni , S. Fu , Y. Yuan , L. Ma , Y. Jiang , L. Li , J. Lu , Adv. Mater. 2018, 30, 1704337. 10.1002/adma.201704337 29314265

[26] L. Yu , J. Liu , X. Xu , L. Zhang , R. Hu , J. Liu , L. Ouyang , L. Yang , M. Zhu , ACS Nano 2017, 11, 5120. 28471641 10.1021/acsnano.7b02136

[27] Y. Xu , E. M. Lotfabad , H. Wang , B. Farbod , Z. Xu , A. Kohandehghan , D. Mitlin , Chem. Commun. 2013, 49, 8973. 10.1039/c3cc45254a 23962906

[28] D. Su , S. Dou , G. Wang , Chem. Mater. 2015, 27, 6022.

[29] L. Ling , Y. Bai , Y. Li , Q. Ni , Z. Wang , F. Wu , C. Wu , ACS Appl. Mater. Interfaces 2017, 9, 39432. 29064226 10.1021/acsami.7b13927

[30] Y. Fu , Q. Wei , X. Wang , H. Shu , X. Yang , S. Sun , J. Mater. Chem. A 2015, 3, 13807.

[31] X. Zhang , H. Chen , Y. Xie , J. Guo , J. Mater. Chem. A 2014, 2, 3912.

[32] W. Ren , W. Zhou , H. Zhang , C. Cheng , ACS Appl. Mater. Interfaces 2017, 9, 487. 27966859 10.1021/acsami.6b13179

[33] X. Wang , Q. Xiang , B. Liu , L. Wang , T. Luo , D. Chen , G. Shen , Sci. Rep. 2013, 3, 2007. 23774372 10.1038/srep02007 PMC3684812

[34] H.‐E. Wang , X. Zhao , X. Li , Z. Wang , C. Liu , Z. Lu , W. Zhang , G. Cao , J. Mater. Chem. A 2017, 5, 25056.

[35] G. Huang , F. Zhang , X. Du , J. Wang , D. Yin , L. Wang , Chem. ‐ Eur. J. 2014, 20, 11214. 25044261 10.1002/chem.201403148

[36] Z.‐T. Shi , W. Kang , J. Xu , Y.‐W. Sun , M. Jiang , T.‐W. Ng , H.‐T. Xue , D. Y. W. Yu , W. Zhang , C.‐S. Lee , Nano Energy 2016, 22, 27.

[37] J. Zhang , M. Li , Z. Feng , J. Chen , C. Li , J. Phys. Chem. B 2006, 110, 927. 16471625 10.1021/jp0552473

[38] R. Wei , X. Tian , Z. Hu , H. Zhang , T. Qiao , X. He , Q. Chen , Z. Chen , J. Qiu , Opt. Express 2016, 24, 25337. 27828472 10.1364/OE.24.025337

[39] X. Wang , K. Chen , G. Wang , X. Liu , H. Wang , ACS Nano 2017, 11, 11602. 29049876 10.1021/acsnano.7b06625

[40] C. Zhao , C. Yu , M. Zhang , Q. Sun , S. Li , M. Norouzi Banis , X. Han , Q. Dong , J. Yang , G. Wang , X. Sun , J. Qiu , Nano Energy 2017, 41, 66.

[41] Z. Xu , H. Wang , Z. Li , A. Kohandehghan , J. Ding , J. Chen , K. Cui , D. Mitlin , J. Phys. Chem. C 2014, 118, 18387.

[42] S. Lei , X. Wang , B. Li , J. Kang , Y. He , A. George , L. Ge , Y. Gong , P. Dong , Z. Jin , G. Brunetto , W. Chen , Z. Lin , R. Baines , D. S. Galvão , J. Lou , E. Barrera , K. Banerjee , R. Vajtai , P. Ajayan , Nat. Nanotechnol. 2016, 11, 465. 26828848 10.1038/nnano.2015.323

[43] Q. Xiao , J. Zhang , C. Xiao , Z. Si , X. Tan , Sol. Energy 2008, 82, 706.

[44] Y. Luo , J. Luo , J. Jiang , W. Zhou , H. Yang , X. Qi , H. Zhang , H. J. Fan , D. Y. W. Yu , C. M. Li , T. Yu , Energy Environ. Sci. 2012, 5, 6559.

[45] D. Wang , Y. Wang , X. Li , Q. Luo , J. An , J. Yue , Catal. Commun. 2008, 9, 1162.

[46] X. Sun , C. Brückner , Y. Lei , Nanoscale 2015, 7, 17278. 26445399 10.1039/c5nr05549k

[47] Y.‐Y. Chen , Y. Zhang , W.‐J. Jiang , X. Zhang , Z. Dai , L.‐J. Wan , J.‐S. Hu , ACS Nano 2016, 10, 8851. 27617483 10.1021/acsnano.6b04725

[48] L. Pan , X.‐D. Zhu , X.‐M. Xie , Y.‐T. Liu , Adv. Funct. Mater. 2015, 25, 3341.

[49] X. Xu , J. Liu , J. Liu , L. Ouyang , R. Hu , H. Wang , L. Yang , M. Zhu , Adv. Funct. Mater. 2018, 28, 1707573.

[50] J. Wang , J. Polleux , J. Lim , B. Dunn , J. Phys. Chem. C 2007, 111, 14925.

[51] W. Duan , Z. Zhu , H. Li , Z. Hu , K. Zhang , F. Cheng , J. Chen , J. Mater. Chem. A 2014, 2, 8668.

[52] Y. Fang , L. Xiao , X. Ai , Y. Cao , H. Yang , Adv. Mater. 2015, 27, 5895. 26305519 10.1002/adma.201502018

