# Supplementary material for: TiO2‐Coated Interlayer‐Expanded MoSe2/Phosphorus‐Doped Carbon Nanospheres for Ultrafast and Ultralong Cycling Sodium Storage
Source: Adv Sci (Weinh). 2018 Nov 9;6(1):1801222. doi: 10.1002/advs.201801222 (PMC6325630; doi:10.1002/advs.201801222)
Supplement: Supplementary file 1 — Supplementary [file ADVS-6-1801222-s001.pdf]

Copyright WILEY-VCH Verlag GmbH & Co. KGaA, 69469 Weinheim, Germany, 2018.

Supporting Information

for *Adv. Sci.,* DOI: 10.1002/advs.201801222

TiO_2-Coated Interlayer-Expanded $MoSe_2$/Phosphorus-Doped Carbon Nanospheres for Ultrafast and Ultralong Cycling Sodium Storage

Yuyu Wang, Yunxiao Wang, Wenpei Kang, Dongwei Cao, Chenxu Li, Dongxu Cao, Zixi Kang, Daofeng Sun,* Rongming Wang, and Yuliang Cao**

Supporting Information

TiO_2-Coated Interlayer-Expanded $MoSe_2$/Phosphorus-Doped Carbon Nanospheres for Ultrafast Sodium Storage and Ultralong Cycling

Yuyu Wang, Yunxiao Wang, Wenpei Kang, Dongwei Cao, Chenxu Li, Dongxu Cao, Zixi Kang, Daofeng Sun,* Rongming Wang, Yuliang Cao**

Y. Y. Wang, Dr. W. P. Kang, D. W Cao, C. X. Li, D. X. Cao, Dr. Z. X. Kang, Prof. D. F. Sun, Prof. R. M. Wang
College of Science, School of Materials Science and Engineering
China University of Petroleum (East China)
Qingdao, Shandong 266580, P. R. China
E-mail: wpkang@upc.edu.cn; dfsun@upc.edu.cn
Dr. Y. X. Wang, Prof. Y. L. Cao
College of Chemistry and Molecular Sciences, Hubei Key Laboratory of Electrochemical Power Sources
Wuhan University
Wuhan, 430072, P. R. China
E-mail: ylcao@whu.edu.cn

The TGA results analysis:

First, the carbon content of $MoSe_2$/P-C should be calculated in order to determine the mole ratio of Mo: C. We assume the mole ratio is m.

$$MoSe_2/P\text{-}C + O_2 \rightarrow MoO_3$$

$$254+12m \qquad 144$$

Through this process, the weight loss ratio can be determined through the following formula:

$$\frac{253.88+12m-143.96}{253.88+12m}$$

And the weight loss was measured to be 52.8% through TG data.

The following equation can be given:

$$\frac{253.88+12m-143.96}{253.88+12m}=52.8\%$$

$$m \approx 4.3$$

The carbon content in $MoSe_2$/P-C is determined to be 16.76%.

The mole ratio of TiO_2 : $MoSe_2$ in $MoSe_2$/P-C@TiO_2 is assumed to be x, the formula weight can be given as follows:

$$MoSe_2/P\text{-}C@TiO_2 + O_2 \rightarrow MoO_3 + TiO_2$$

$$253.88 + 79.87x + 12 \times 4.3 \quad 143.96 \quad 79.87x$$

Based on the weight loss from the TG result, an equation can be given:

$$\frac{253.88+79.87x+12\times4.3-143.96-79.87x}{253.88+79.87x+12\times4.3}=47.4\%$$

$$x = 0.45$$

So the formula weight of $MoSe_2$/P-C@TiO_2 is (253.88 + 79.87 × 0.45 + 12 ×4.3)

And the contents of TiO_2, $MoSe_2$ and carbon in the composite are calculate to be 10.53%, 74.36% and 15.11%, respectively.

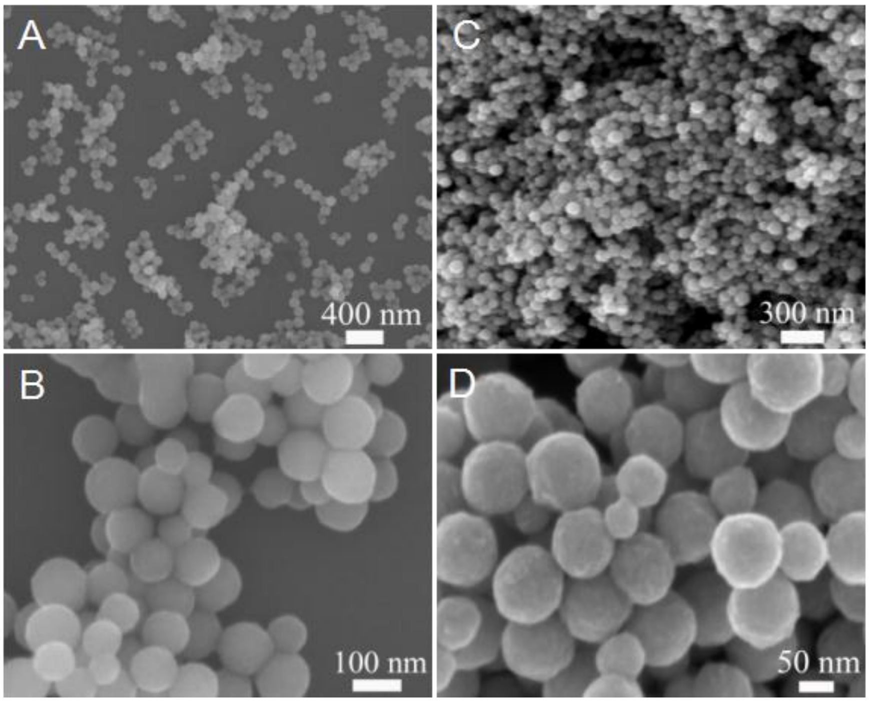

Figure S1. FESEM images of A,B) PPy-PMo_{12} precursor, C,D) $MoSe_2$/P-C at different magnifications.

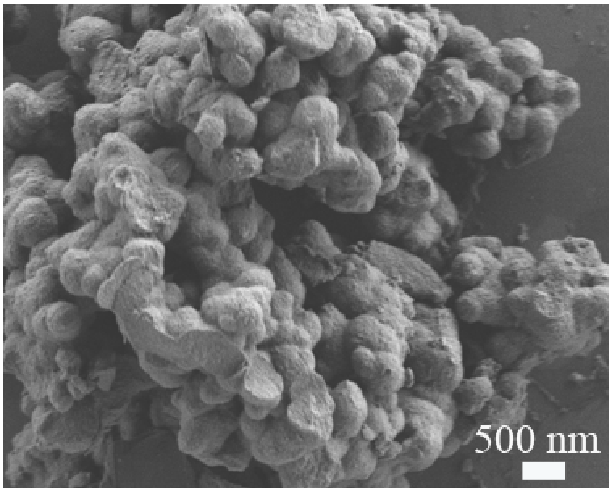

Figure S2. FESEM image of $MoSe_2$@TiO_2.

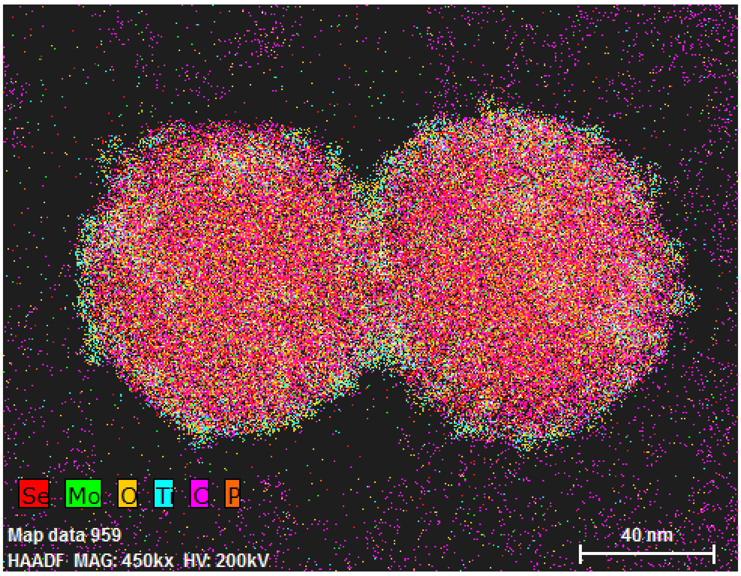

Figure S3. The corresponding EDX mapping image showing the distribution of Mo, Se, Ti, O, C, and P for $MoSe_2$/P-C@TiO_2 nanospheres.

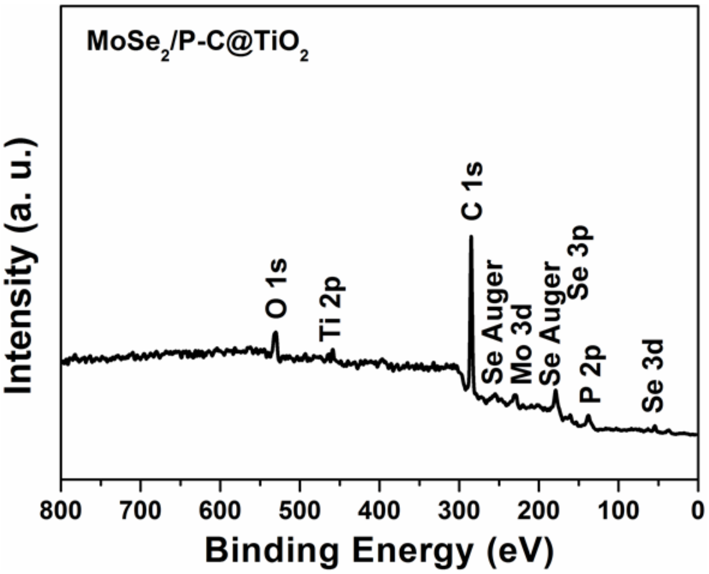

Figure S4. Survey spectrum of the $MoSe_2$/P-C@TiO_2 composite.

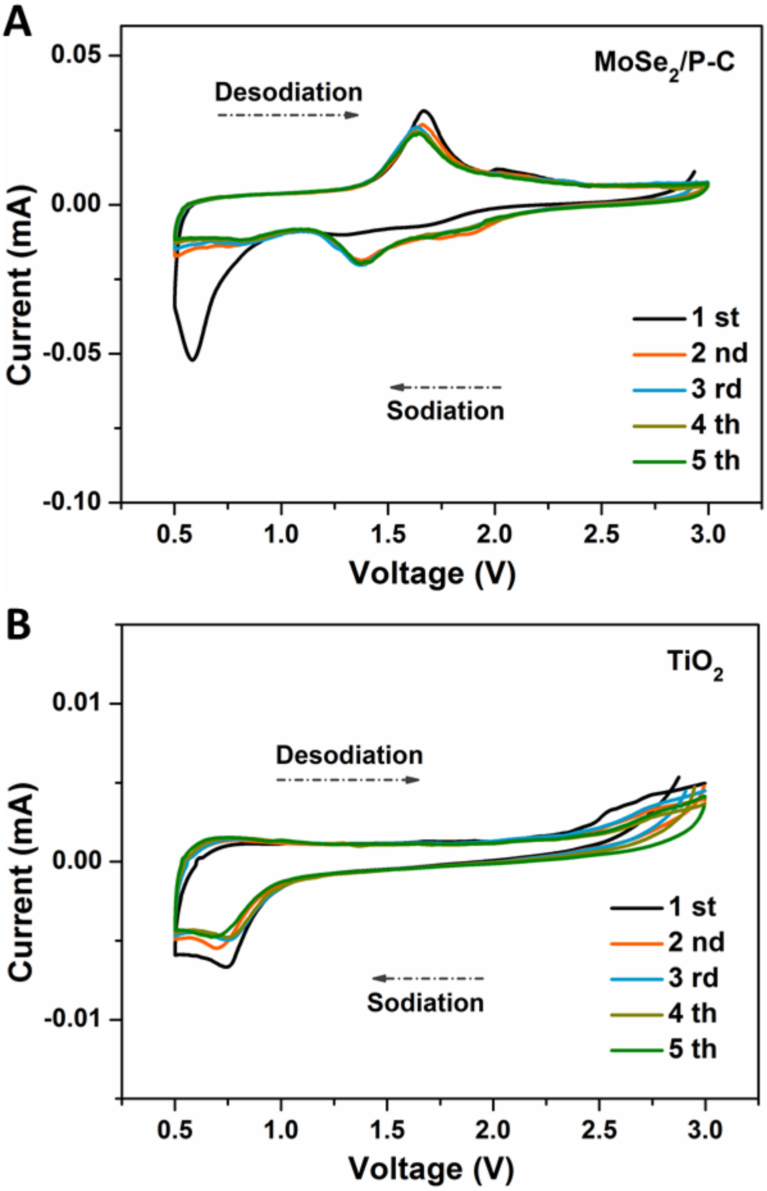

Figure S5. CV curves of A) $MoSe_2$/P-C electrode, B) TiO_2 electrode.

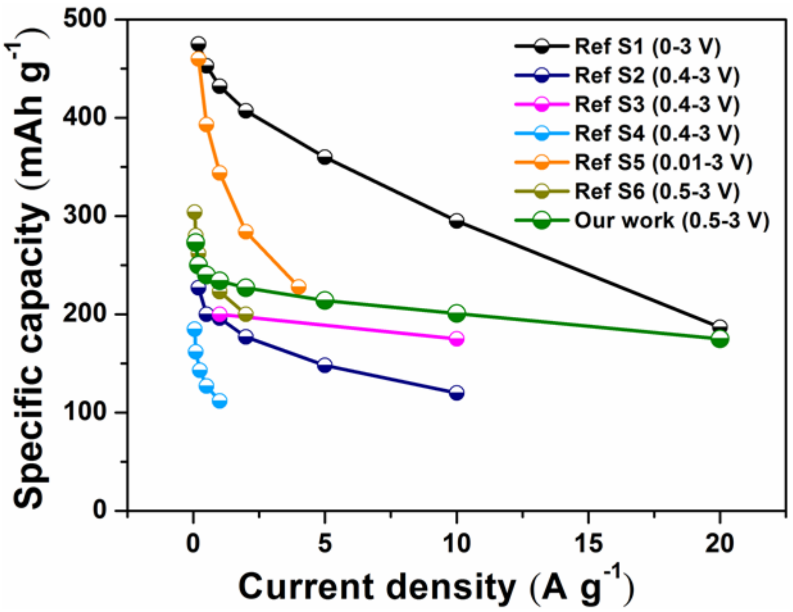

Figure S6. Rate capability comparison of other MoS_2/$MoSe_2$-based nanostructures as anode materials for SIBs.

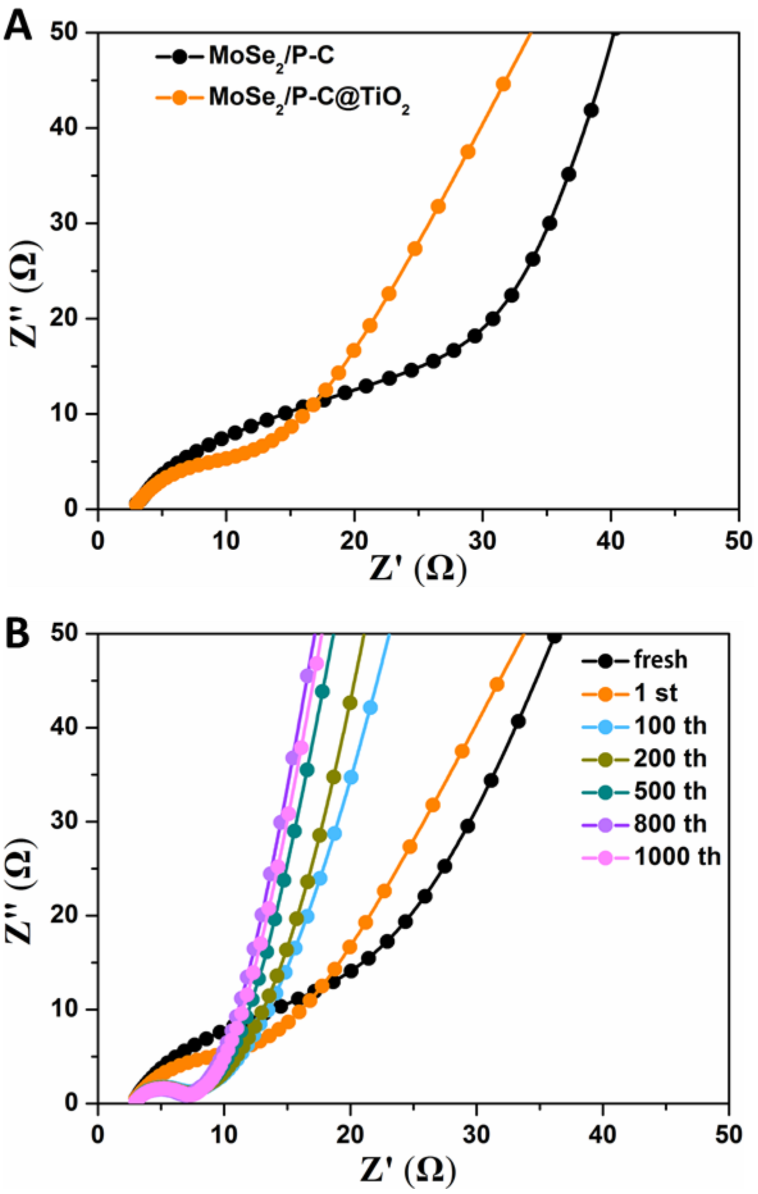

Figure S7. Nyquist plots of A) the $MoSe_2$/N,P-C@TiO_2 composite and $MoSe_2$/P-C electrodes after 1 cycle, and B) the $MoSe_2$/N,P-C@TiO_2 composite electrode after different cycles.

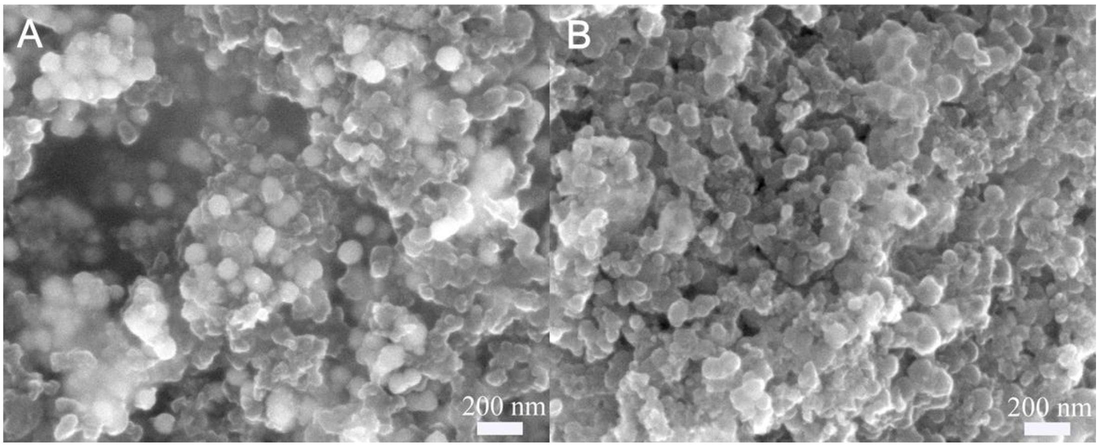

Figure S8. SEM images of $MoSe_2$/N,P-C@TiO_2 composite electrode A) before charge-discharge cycle, B) after 1000 charge-discharge cycles.

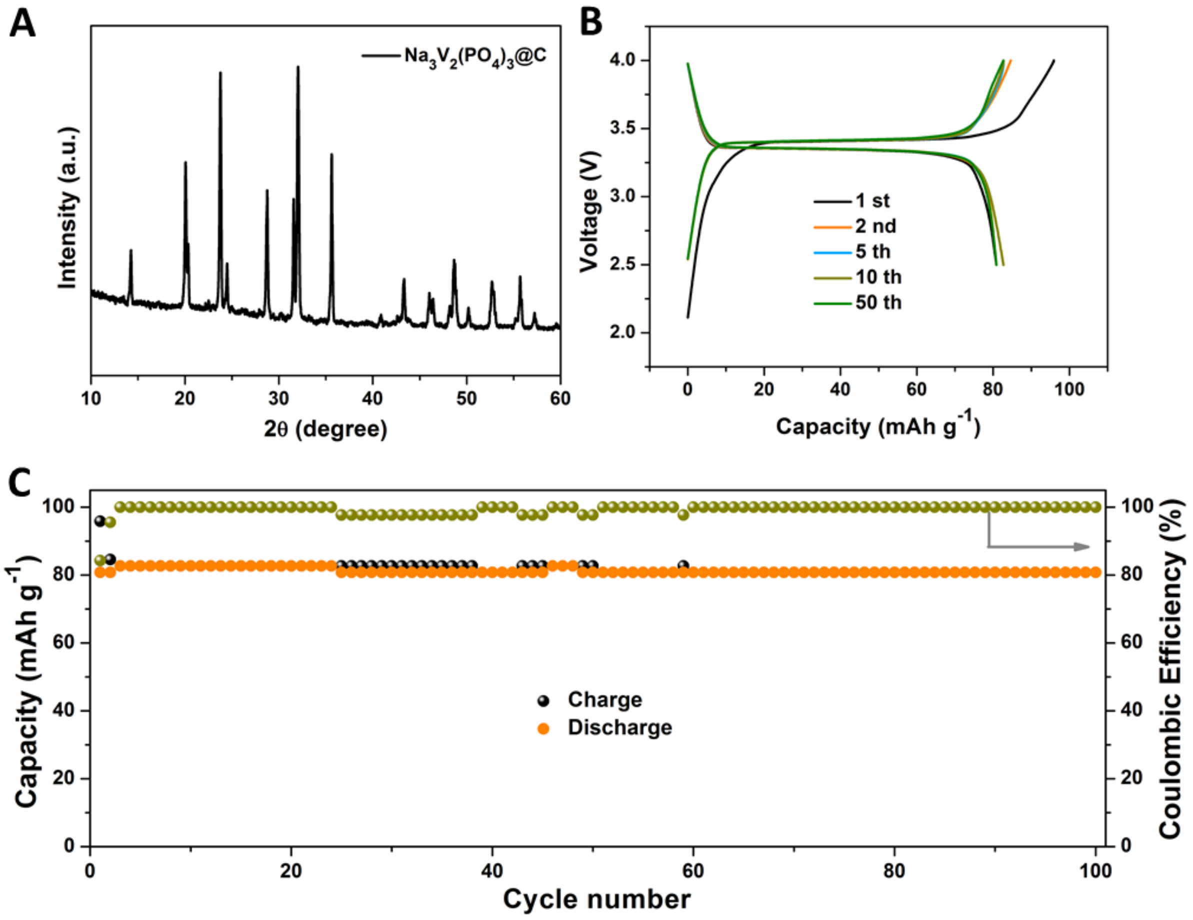

Figure S9. A) XRD pattern of the homemade NVP@C, B) Galvanostatic charge and discharge curves, C) cycling performance of NVP@C half cells at 200 mA g^{-1}.

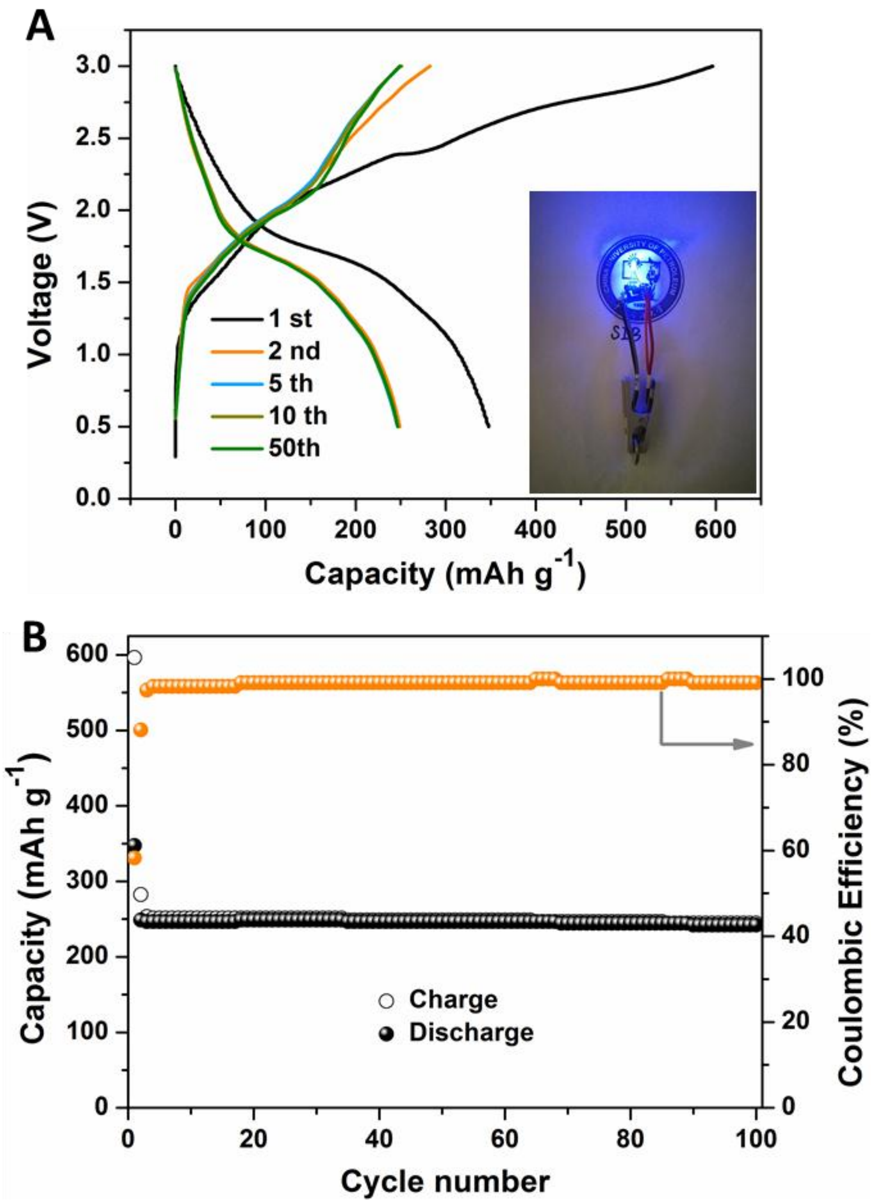

Figure S10. A) Charge-discharge curves of $MoSe_2$/P-C@TiO_2//NVP@C full cell at 500 mA g^{-1} with a voltage window of 0.5-3.0 V; the inset digital image shows a blue LED light powered by the current full cell, B) Cycling performance of $MoSe_2$/N,P-C@TiO_2//NVP@C full cell at 500 mA g^{-1} and 0.05 A g^{-1} in the first cycle.

[S1] Z.-T. Shi, W. Kang, J. Xu, Y.-W. Sun, M. Jiang, T.-W. Ng, H.-T. Xue, D. Y. W. Yu, W. Zhang, C.-S. Lee, *Nano Energy* **2016**, *22*, 27.

[S2] Y.-L. Ding, P. Kopold, K. Hahn, P. A. van Aken, J. Maier, Y. Yu, *Adv. Mater.* **2016**, *28*, 7774.

[S3] Z. Hu, L. Wang, K. Zhang, J. Wang, F. Cheng, Z. Tao, J. Chen, *Angew. Chem., Int. Ed.* **2014**, *126*, 13008.

[S4] Y. Li, Y. Liang, F. C. R. Hernandez, H. Deog Yoo, Q. An, Y. Yao, *Nano Energy* **2015**, *15*, 453.

[S5] D. Xie, W. Tang, Y. Wang, X. Xia, Y. Zhong, D. Zhou, D. Wang, X. Wang, J. Tu, *Nano Res.* **2016**, *9*, 1618.

[S6] J. Zhang, M. Wu, T. Liu, W. Kang, J. Xu, *J. Mater. Chem. A* **2017**, *5*, 24859.
